# Increased augmentation index in patients with Ehlers-Danlos syndrome

**DOI:** 10.1186/s12872-020-01684-x

**Published:** 2020-09-15

**Authors:** Maurice Roeder, Sira Thiel, Frederic Baumann, Noriane A. Sievi, Marianne Rohrbach, Malcolm Kohler, Thomas Gaisl

**Affiliations:** 1 Department of Pulmonology, University Hospital Zurich, Rämistrasse 100, 8091 Zurich, Switzerland; 2 Clinical and Interventional Angiology, University Hospital Zurich, Zurich, Switzerland; 3 Division of Metabolism and Children’s Research Center University Children’s Hospital Zurich, Zurich, Switzerland; 4 Centre for Interdisciplinary Sleep Research, University of Zurich, Zurich, Switzerland

**Keywords:** Ehlers-Danlos syndrome, Arterial stiffness, Augmentation index, Cardiovascular risk

## Abstract

**Background:**

Ehlers-Danlos Syndrome (EDS) comprises a heterogeneous group of diseases characterized by joint hypermobility, connective tissue friability, and vascular fragility. Reliable prognostic factors predicting vascular disease progression (e.g. arterial aneurysms, dissections, and ruptures) in EDS patients are still missing. Recently, applanation tonometry derived augmentation index (AIx), an indirect marker of arterial stiffness, has shown to be positively associated with progression of aortic disease in Marfan syndrome. In this study, we assessed aortic AIx in patients with EDS and matched healthy controls.

**Methods:**

We performed noninvasive applanation tonometry in 61 adults with EDS (43 women and 18 men aged 39.3 ± 14.6 years) and 61 age-, gender-, height-, and weight-matched healthy controls. Radial artery pulse waveforms were recorded and analyzed using the SphygmoCor System (AtCor Medical, Sydney, NSW, Australia). Calculated AIx was adjusted to a heart rate of 75/min. Groups were compared and association between AIx and EDS was determined by univariate and multivariate regression analysis.

**Results:**

EDS patients were categorized in classical type EDS (34%), hypermobile type EDS (43%), vascular type EDS (5%), or remained unassignable (18%) due to overlapping features. EDS patients showed a significantly increased aortic AIx compared to healthy controls (22.8% ± 10.1 vs 14.8% ± 14.0, *p* < 0.001). EDS showed a positive association with AIx; independent of age, sex, height, blood pressure, medication, and pack years of smoking.

**Conclusions:**

Patients with EDS showed elevated AIx, indicating increased arterial stiffness when compared to healthy controls. Further investigations are needed in order to assess the prognostic value of increased AIx for cardiovascular outcomes in patients with EDS.

## Background

Ehlers-Danlos Syndrome (EDS) represents a heterogeneous group of heritable connective tissue disorders characterized by joint hypermobility, connective tissue friability, as well as skin and vascular fragility. The combined prevalence of all EDS subtypes appears to be nearly 1 to 5000 individuals world- wide [1]. The current international classification of EDS distinguishes 13 clinical subtypes, for which molecular mutations have been identified in 19 different genes [2].

Vascular fragility with easy bruising is common in all patients with EDS, but its severity varies between EDS subgroups [3]. An increased bleeding tendency including menometrorrhagia, gum bleeding, and peri-operative hemorrhage has been described in many EDS-patients of varying EDS subtypes [3]. Spontaneous aneurysms, dissections and ruptures of medium to large sized arteries, caused by defects in type III collagen, are a clinical hallmark of the vascular type of EDS (vEDS). These life-threatening complications result in a reduced life span with a median age of approximately 51 years [4]. Major vascular events have also been described in EDS subtypes with other molecular defects, including defects of collagen I and V or molecules involved in collagen folding (FKBP22), processing (ADAMTS-2) or modification (LH1) [3].

Recent advances in surgical and endovascular management of progressive arterial disease in EDS patients have been associated with good clinical outcomes [5–8]. Nevertheless, due to the difficult handling of fragile tissue and vessels during the operation, a sufficient risk-benefit assessment becomes crucial. Given that spontaneous arterial rupture can also occur in non-dilated arteries, prognostic factors for identification of vascular high-risk patients are indispensable, yet undetermined to date [9].

Arterial tonometry derived augmentation index (AIx) is measured by pulse wave analysis, and is an indirect surrogate measure of arterial stiffness [10]. It has been shown to be an independent predictor of mortality and cardiovascular events in patients with hypertensive, cardiovascular and renal disease [11]. Furthermore, recent findings suggest that the AIx is elevated in patients with Marfan syndrome and correlates with progression of aortic disease [12, 13]. Therefore, in a first step, the AIx of EDS patients was compared to age-, gender-, weight-, and height-matched healthy controls and possible predicting factors were assessed.

## Methods

### Study design and participants

This study was part of a recently published prospective parallel-cohort study, which our group conducted in order to assess the prevalence of obstructive sleep apnoea in patients with EDS [14]. For this purpose, 100 adult patients with EDS were one-to-one matched to 100 healthy adult controls according to sex, age, weight and height. Study procedures included structured interviews (including short-form 36), level-3 respiratory polygraphy and arterial tonometry.

Between April and December 2015, EDS patients have been recruited from three different sources: University Children’s Hospital Zurich EDS database, University Hospital Zurich EDS database, and an international network based on EDS associations. EDS diagnosis and categorization followed the Villefranche diagnostic criteria [15]. Hypermobility was assessed according to the Beighton score [16]. EDS diagnosis was confirmed either by 1) gene analysis, 2) pathological electron microscope study findings or biochemical analysis of collagen in cultivated fibroblasts suggestive for specific EDS subtypes or 3) increased ratio of deoxypyridinoline to pyridioline crosslinks in biochemical urine analysis for kyphoscoliotic EDS (kEDS: formerly EDS VIA) [17–19]. Healthy control subjects were recruited from the local population in Zurich to specifically match (one-to-one matching) the patients with EDS in terms of sex, age (± 3 years), height (±20 cm) and weight (±15 kg). If subjects were aged 18 years or above, physically and intellectually capable to adhere to the study protocol, and were not pregnant, we considered them eligible. 61 patients with EDS agreed to the applanation tonometry at the study site in Zurich and thus were included in the study. Accordingly, 61 matched healthy controls were also investigated and included in this study. All participants provided written informed consent to participate in the study. All procedures were reviewed and approved by the Cantonal Ethics Committee Zurich (registration number KEK-ZH- 2015-0144) and conformed to the Declaration of Helsinki.

### Measurements

#### Pulse wave analysis

Before pulse wave analysis, subjects had to rest ten minutes in supine position. Subsequently, radial artery pulse waveforms were recorded using the SphygmoCor System (AtCor Medical, Sydney, NSW, Australia). Approximately ten radial pulse waves were measured and a corresponding central aortic pressure waveform was generated using a validated mathematical transfer function [20]. The inflection point of the aortic pressure waveform corresponds to the onset of the reflected wave returning from peripheral arteries and divides the aortic pressure wave into an early and late systolic peak. This inflection point can be determined with an algorithm. The AIx quantifies the augmentation of central aortic pressure, hence representing a measure of peripheral arterial wave reflection. AIx can be calculated as the difference between the second (P2) and the first systolic peak pressure (P1) and is expressed as percentage of central pulse pressure (PP):

$$ \mathrm{AIx}\ \left(\%\right)=\left[\left(\mathrm{P}2-\mathrm{P}1\right)/\mathrm{PP}\right]\times 100. $$

Since AIx is influenced by heart rate [21, 22], the index is adjusted to a heart rate of 75 bpm. SphygmoCor Px software adjusts the AIx at an inverse rate of 4.8% for each 10 bpm increment. To ensure a high measurement quality, only measurements with an operator index of 80 and above were accepted. The measurements were conducted by two trained, unblinded researchers.

### Data analysis and statistics

All data are presented as mean ± standard deviation (SD) or median (quartiles) unless otherwise stated. Groups were compared by independent t-test, Wilcoxon rank sum test or ANOVA. Univariate regression analysis was used to investigate association between AIx and EDS as well as possible and known (prespecified) predictors for AIx such as age, sex, height, weight, BMI, blood pressure (BP), smoking status, and use of medication. To further investigate the independent association between AIx and EDS, multivariate analysis was performed including all variables showing *p*-value < 0,1 in univariate regression analysis. We assessed eight possible clinical predictors with multiple regression analysis. Using a probability level of 0.05 the observed R^2^ of 0.6 in the final model led to a statistical power of 100% [23, 24]. Residual analysis of the model was performed to check the regression assumption. A two-sided *p*-value of < 0,05 was considered statistically significant.

## Results

### Study participants and baseline characteristics

**Fig. 1:**
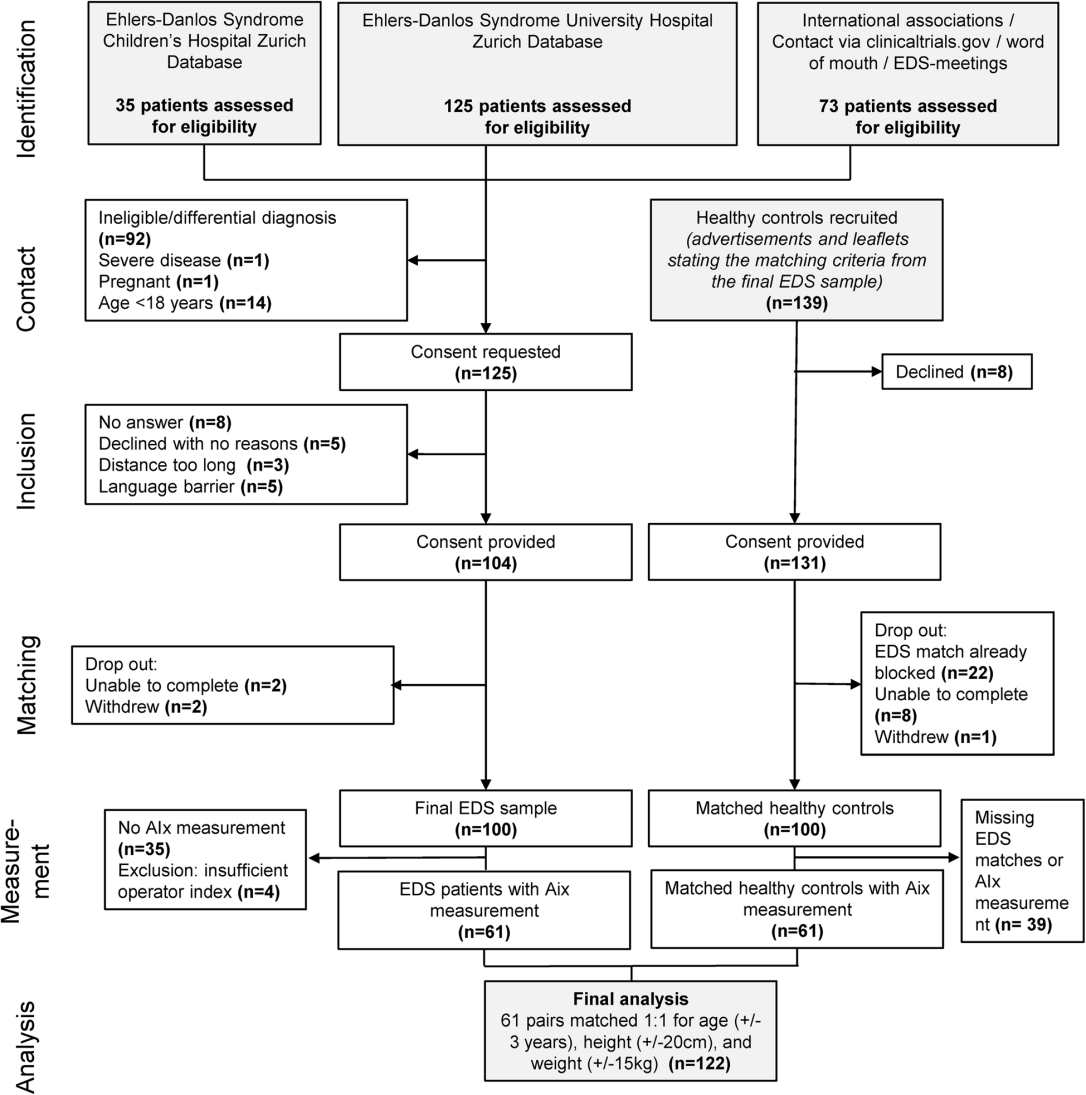
Study flow chart. The majority of the study participants were recruited by hospital-wide screening of electronic databases (Children’s Hospital Zurich and University Hospital Zurich). 200 study participants were enrolled, and 122 participants were included in the final analysis. EDS, Ehlers-Danlos syndrome. AIx, Augmentation Index

A total of 65 EDS patients underwent pulse wave analysis. Due to insufficient measurement quality, four subjects had to be excluded. Subsequently, 61 healthy controls were matched. A total of 122 subjects entered the final analysis of pulse wave analysis measurement (Fig. 1). EDS Patients were categorized in classical type EDS (34%), hypermobile type EDS (43%), vascular type EDS (5%) or remained unassignable (18%) due to overlapping features although Villefranche inclusion criteria were fulfilled. AIx measurements took place after mean (SD) 10 ± 13.1 years after the initial EDS diagnosis. The mean (SD) age in the EDS cohort and the healthy controls was 39.3 ± 14.6 years and 35.6 ± 11.4 years, respectively. Diastolic BP (80.6 ± 10.8 mmHg vs 76.1 ± 11.8 mmHg) and the number of patients using antihypertensive drugs (16% vs 2%) or NSAIDs (21% vs 3%) was significantly higher in the EDS cohort. The most important class of antihypertensive drugs in EDS patients was ACE inhibitor/ATII receptor blocker (7 out of 10 patients), while one patient of the control group used a beta-blocker. The detailed patients’ characteristics are shown in Tables 1 and 2.

**Table 1:** Baseline characteristics of the 122 one-to-one matched study participants

	EDS cohort (*n* = 61)	Healthy control cohort (n = 61)	***p***-value
Sex (f / m)	43 / 18	43 / 18	–
Caucasian, %	100	100	–
Age, years	39.3 ± 14.6	35.6 ± 11.4	0.13
Height, cm	168.5 (161.0/174.5)	169.0 (162.5/176.5)	0.47
Weight, cm	65.0 (54.0/76.0)	65.0 (58.0/75.5)	0.73
BMI, kg/m^2^	22.7 (20.4/25.9)	22.9 (20.8/24.7)	0.96
BSA, m^2^	1.3 (1.1/1.5)	1.3 (1.2/1.5)	0.81
Neck, cm	33 (31/37)	33 (32/37)	0.70
Waist, cm	73 (67/85)	76 (69.0/81.0)	0.87
Blood pressure systolic (office), mmHg	117.4 ± 14.2	112.8 ± 12.8	0.07
Blood pressure diastolic (office), mmHg	80.6 ± 10.8	76.1 ± 11.8	0.03
Pulse, min^−1^	76.7 (67/84.7)	69.0 (64.0/76.7)	0.025
Alcohol units per week, units	0 (0/1)	0 (0/2)	0.74
Current Smoker, n (%)	15 (25%)	9 (15%)	0.20
Diabetes, n (%)	3 (4.9%)	0 (0%)	0.08
Arterial hypertension, n (%)	10 (16%)	1 (2%)	0.004
Pack years of smoking, n	0 (0/3)	0 (0/1)	0.11
Antihypertensive drugs, n (%)	10 (16%)	1 (2%)	0.004
NSAIDs, n (%)	13 (21%)	2 (3%)	0.002
Lipid lowering drugs, n (%)	0	0	–
Antiplatelet drugs, n (%)	1 (1.64%)	0	0.32
Antidiabetic drugs, n (%)	0	0	–

*EDS* Ehlers-Danlos Syndrome; *BMI* Body-Mass-Index; *BSA* body surface area; *NSAID* non-steroidal anti-inflammatory drugs

**Table 2:** Aortic Augmentation Index @HR 75 in different EDS subtypes

EDS type	Classical type	Hypermobile type	Vascular type	Not assignable^a^	***p***-value
N	21 (34%)	26 (43%)	3 (5%)	11 (18%)	–
Beighton score	5.7 ± 2.5	5.4 ± 2.5	2.3 ± 1.5	5.5 ± 2.5	0.199
Aortic Augmentation Index @HR 75, %	22 ± 11	22 ± 11	32 ± 10	25 ± 11	0.451

^a^Not clearly attributable due to overlapping features, although Villefranche inclusion criteria are fulfilled. Patients fulfilling diagnostic criteria for more than one EDS subtype were labeled “not assignable” for this study. *EDS* Ehlers-Danlos Syndrome; *HR* heart rate

### Pulse wave analysis

**Fig. 2:**
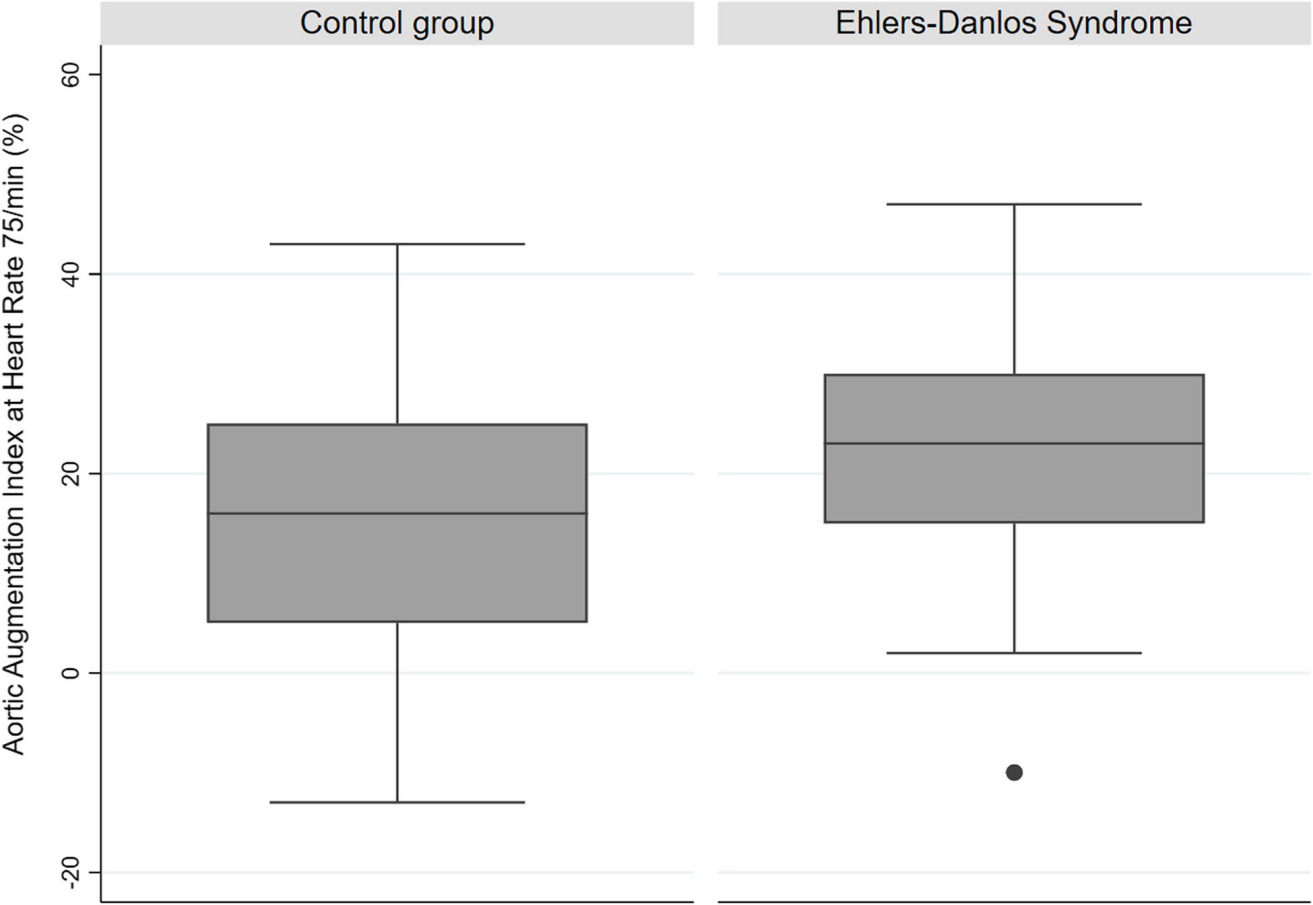
Box plots for aortic augmentation index adjusted to heart rate of 75/min by groups

EDS patients showed a significantly increased aortic AIx compared to healthy controls (22.8 ± 10.1% vs 14.8 ± 14.0%, *p* < 0.001) as shown in Fig. 2. The primary wave pressure (P1 height) showed no statistical difference (median (quartiles) 20 (16/23) mmHg vs 20 (17/24) mmHg). While the time to the first aortic pressure peak (T1) was comparable, the time to the second aortic pressure peak (T2) and the ejection duration (ED) was significantly shorter in the EDS cohort. EDS patients had a significantly higher resting heart rate during the pulse wave analysis. Patients suffering vascular type EDS showed the highest AIx (mean (SD) 31.2 ± 14.1%), even though there was no statistical difference between the assessed EDS subtypes. Detailed information about the results of pulse wave analysis are shown in Table 3.

**Table 3:** Pulse wave analysis in both cohorts

	EDS cohort (n = 61)	Healthy control cohort (n = 61)	***p***-value
Aortic AIx (AP/PP) @HR 75, %	22.8 ± 10.1	14.8 ± 14.0	< 0.001
P1 height, mmHg	20 (16/23)	20 (17/24)	0.438
Peripheral T1, ms	113 (104/122)	109 (104/119)	0.288
Peripheral T2, ms	200 (191/212)	209 (202/221)	0.002
Peripheral AIx, %	80.4 ± 18.2	72.5 ± 17.2	0.017
End systolic pressure, mmHg	102 (92/109)	97 (91/105)	0.103
Ejection duration, ms	296 (276/309)	302 (288/316)	0.025
Heart rate, min^−1^	76 (67/83)	67 (63/72)	< 0.001
Mean pressure systolic, mmHg	99 (91/107)	96 (90/102)	0.142
Mean pressure diastolic, mmHg	89 (82/96)	85 (79/91)	0.069

*AIx* Augmentation index; *EDS* Ehlers-Danlos Syndrome; *HR* heart rate

### Predictors of aortic AIx

AIx was positively associated with age, diastolic BP, pack years of smoking, and use of antihypertensive drugs. Male sex, weight and height were negatively associated with AIx in univariate regression analysis (Table 4). Table 5 presents the independent predictors of AIx analyzed by a multiple regression model. EDS showed a significant, positive, and independent association with AIx (Coef. (95% CI) of 3.85 (0.41 to 7.29), *p* = 0.029). While age, diastolic BP and pack years of smoking were independently associated with AIx, the use of medication showed no independent association in the multiple regression analysis. Height and male sex showed a negative, independent association with AIx. Due to strong correlation between height and weight (r = 0.70, *p* < 0.001), weight was removed from the final model.

**Table 4:** Univariate regression analysis of possible predictors for aortic AIx (AP/PP) normalized for a heart rate of 75/min in all study subjects (*n* = 122)

Variable	Coefficient	95% Confidence Interval	***p***-value
Age, years	0.43	0.27 to 0.59	**< 0.001**
Male sex, (yes/no)	−13.36	−17.96 to −8.76	**< 0.001**
Height, cm	−0.61	−0.80 to −0.42	**< 0.001**
Weight, kg	−0.21	− 0.36 to − 0.06	**0.007**
BMI, kg/m^2^	0.19	−0.40 to 0.78	0.532
Ehlers-Danlos Syndrome, (yes/no)	8.10	3.59 to 12.60	**0.001**
Mean systolic blood pressure, mmHg	0.124	−0.05 to 0.30	0.168
Mean diastolic blood pressure, mmHg	0.42	0.22 to 0.61	**< 0.001**
Smoking, (yes/no)	2.78	−3.18 to 8.79	0.357
Pack years of smoking, n	0.41	0.12 to 0.69	**0.006**
Antihypertensive Drugs, (yes/no)	8.67	0.55 to 16.79	**0.037**
Antiplatelet Drugs, (yes/no)	−5.88	−32.13 to 20.37	0.658
NSAID, (yes/no)	7.03	−0.07 to 14.19	0.052

*AIx* Augmentation index; *BMI* Body-Mass-Index; *NSAID* non-steroidal anti-inflammatory drugs

**Table 5:** Multivariate linear regression analysis of possible predictors for aortic AIx (AP/PP) normalized for a heart rate of 75/min in all study subjects (n = 122)

Variable	Coefficient	95% Confidence Interval	***p***-value
Ehlers-Danlos Syndrome, (yes/no)	3.85	0.41 to 7.29	**0.029**
Height, cm	−0.43	− 0.64 to − 0.23	**< 0.001**
Male sex, (yes/no)	−8.00	−12.48 to −3.52	0.001
Age, years	0.32	0.18 to 0.46	**< 0.001**
Pack years of smoking, n	0.26	0.05 to 0.47	**0.015**
Mean diastolic blood pressure, mmHg	0.16	0.01 to 0.31	**0.042**
Antihypertensive drugs, (yes/no)	3.75	−1.82 to 9.33	0.185
NSAID, (yes/no)	1.00	−3.99 to 5.99	0.692

*AIx* Augmentation index; *NSAID* non-steroidal anti-inflammatory drugs

## Discussion

This study investigated the applanation tonometry derived augmentation index (AIx) in a well characterized cohort of EDS patients. EDS patients showed a significantly, independently increased AIx compared to matched healthy controls, suggesting increased arterial stiffness in EDS patients despite the known influencing factors such as age, sex, BP and smoking history. Although vascular EDS patients showed the highest AIx (31.2 ± 14.1%), no significant overall differences of the AIx could be observed between the different types of EDS.

The aortic AIx is used as an indirect index of vascular stiffness derived from aortic pressure waveform analysis [25, 26]. It represents the ratio of the ejection pressure from the heart and the reflection pressure from the arterial system. In this study, the primary wave pressure (P1), which represents the primary left ventricular ejection pressure, showed no difference between EDS patients and healthy controls, leading to the conclusion, that the observed difference in AIx results from the arterial reflection wave. These findings suggest that the increased AIx might represent increased arterial stiffness in EDS patients. This hypothesis is supported by the fact that the time of the second aortic peak (T2) was significantly shorter in the EDS cohort, while the time for the first peak (T1) was comparable. The finding of an increased AIx in EDS patients cannot be compared to other studies yet due to missing literature on this topic. However, since the AIx of our healthy controls (14.8 ± 14.0%) are comparable to expected reference values obtained from larger unselected population-based studies the findings in EDS seems to be plausible [27]. Furthermore, AIx was positively correlated to female gender, age, diastolic BP, and pack years of smoking, and was also negatively correlated to height and weight as reported in previous studies [27–30].

Literature about arterial stiffness and cardiovascular risk in EDS patients is rare and the existing results are incongruent due to inhomogeneous study populations and different methods used to determine arterial stiffness [31–35]. Population inhomogeneity is mainly based on the fact that EDS is an umbrella term for clinically diverse connective tissue disorders based on different genetic defects [36].

Our results contradict in particular a recently published study by Miller et al., reporting increased arterial elasticity in patients with EDS measured by pulse wave velocity (PWV) [35]. Nevertheless, this study only retrospectively analyzed a subset of EDS patients from the Clinical and Molecular Manifestations of HDCT study who had both orthostatic BP recordings and PWV measurements [37]. There might be a selection bias, because the authors focused especially on the connection between orthostatic intolerance (OI) and EDS patients. The comparison of these results with our study has to be done with caution, as only few information on study population is available and the EDS patients were not matched to a healthy control group. Nevertheless, we have to point out that AIx is a measure of pressure wave reflection, while PWV is the most commonly and universally acceptable measure of arterial stiffness. The assessment of central/aortic pressures, PWR and AIx in this specific population compared to healthy controls are of great interest and should be the focus of future research.

Vascular complications in patients with EDS are historically attributed to impaired synthesis and processing of diverse collagen types, leading to fragility of blood vessel walls and perivascular connective tissue, thus a decreased arterial stiffness would be suspected. However, molecular pathomechanisms underlying development of arterial aneurysms, dissections, rupture and bleeding are poorly understood [3]. It is to mention that arterial stiffness is not only determined by the arterial wall’s composition of elastin and collagen. For example, smooth muscle cells behave as a stiff material linking fibrous component and contribute to arterial stiffness especially when contracted [38–40]. Furthermore, animal studies suggest that effective arterial stiffness results from complex interactions between smooth muscle cells and extracellular matrix. For instance, increased fibronectin, an extracellular protein, is associated with a cell dedifferentiation process leading to increased production of extracellular matrix proteins by smooth muscle cells [41]. Stewart et al. [42] found that the aortic AIx and carotid-femoral pulse wave velocity increases after the application of a nitric oxide synthase inhibitor, thus pointing out the importance of basal nitric oxide on functional regulation of central arteries in humans. Nitric oxide synthesis could be altered in EDS patients given the recent finding that markers of endothelial dysfunction such as VCAM-1, ICAM-1 and MCP-1 are increased in vEDS patients [43].

Our EDS patients showed increased resting heart rate and BP compared to healthy controls. Increased resting sympathetic activity has already been described as part of autonomic dysregulation in EDS patients [44]. The connection between elevated BP, increased arterial stiffness and vascular disease has been extensively reviewed in the general literature about cardiovascular pathology. Chronic elevated BP leads to decreased vascular distensability that, in turn, increases pulsatile shear stress and pressure leading to endothelial dysfunction and vascular disease [45, 46]. Increased arterial wall stress in EDS patients compared to healthy controls has been reported by Boutouyrie et al. [47] using high-resolution echo-tracking systems.

Shingu et al. [48] found an elevated carotid AIx in patients with aortic aneurysm and dissection, which the authors attributed to increased left ventricular afterload. Whether an increased prevalence of aortic aneurysms and dissections could be a reason for the increased AIx in our EDS cohort remains unknown because study subjects did not undergo vasculopathy evaluation. Depending on EDS subtypes, EDS patients suffer joint pain, joint dislocation, muscle cramps and fatigue leading to significantly reduced physical activity [49]. There are several studies suggesting a link between decreased physical activity and increased arterial stiffness [50, 51]. Even when only considering the number of reported joint luxation in our EDS cohort (22% of all EDS patients), reduced daily activity as a cause for increase arterial stiffness should be considered. In this study, EDS patients used more NSAIDs and antihypertensive medication compared to healthy controls. The influence of several medications, including antihypertensive and anti-inflammatory drugs on arterial stiffness has been described in literature [41], but no independent pharmacological influence on the AIx was observed in our study. Furthermore, it is to mention that no EDS patient was treated with Celiprolol, a cardioselective β-blocker that has recently been shown to increase stiffness of the common carotid artery in vEDS patients [52].

Since medical and interventional management advances lead to improved survival and quality of life in EDS patients, the role of early identification of vascular high-risk patients gains importance. To date, little is known about prognostic factors that could predict the chance of developing fatal cardiovascular events in patients with EDS. Pepin et al. [4] recently showed that the natural course of vEDS varies with gender and type of mutation in COL3A1. This group showed a 5-year survival difference between males and females which was primarily based on a higher death rate before age of 20 due to vascular rupture. Vascular inflammation marker has been shown to be significantly elevated in vascular EDS patients compared to matched controls [53]. However, their potential to predict vascular complications in EDS patients are still subject of ongoing investigation [43].

Increased applanation tonometry derived AIx has been reported in patients with Marfan syndrom [13], a genetic connective tissue disorder due to mutation in the fibrillin 1 gene [54]. These patients also suffer from fatal vascular events like aortic dissection and rupture [55]. Mortensen et al. [13] conducted a longitudinal observational study to follow-up cardiovascular disease progression in 50 adult patients with Marfan syndrome for almost 2 years. Beside baseline aortic root diameter, only aortic AIx showed an independent association to aortic disease progression. Furthermore, Kaplan-Meier curve analysis presented a significantly lower rate of aortic root disease in patients with lower AIx [12]. EDS and Marfan syndrome are multisystemic disorders that primarily affect the soft connective tissues. Even if these disorders differ in genetic and molecular pathogenesis, there are at least some clinical overlaps [36] suggesting that the increased AIx might predict vascular disease progression in EDS patients as reported in Marfan syndrome patients. In addition, the fact that the other “non-vascular” EDS subtypes showed increased AIx as well, could point out an increased risk for cardiovascular events beside the vEDS-typical vascular complications. However, data about the general cardiovascular risk in EDS patients compared to the general population are lacking.

There are some limitations to our study. The patient numbers of EDS subtypes, especially of vEDS, were relatively small to allow statistical comparison of different AIx values between different subtypes. Nonetheless, related to EDS incidence, the number of acquired patients was considerable. Arterial stiffness was not the primary outcome of this study, hence no specific power calculation for pulse wave analysis measurement were done. However, the AIx difference measured in EDS patients and healthy controls was considerably. In addition, our study design did not allow a longitudinal follow-up of cardiovascular events in EDS patients. Further longitudinal studies assessing AIx and cardiovascular events in EDS patients would be needed to show the clinical relevance of increased AIx as a prognostic factor for cardiovascular risk in these patients. Nevertheless, since vEDS patients harbor the highest risk for fatal vascular events and this subgroup presented the highest AIx in our study, AIx already indicate potential as a possible predictor for vascular high-risk patients.

## Conclusion

This study showed increased applanation tonometry derived AIx in patients with EDS suggesting elevated arterial stiffness compared to healthy controls. Additional investigations have to assess the predictive value of increased arterial stiffness regarding general cardiovascular risk in patients with EDS. Furthermore, prospective observational studies are needed in order to evaluate whether AIx might provide similar prediction of aortic disease progression as it was shown in patients with Marfan syndrome.

## Data Availability

The datasets used and/or analysed during the current study are available from the corresponding author on reasonable request.

## Abbreviations

AIx: Augmentation index
AP: Augmentation pressure
BMI: Body mass index
BP: Blood pressure
BSA: Body surface area
Coef.: Coefficient
COL1A1: Collagen type III alpha 1 chain
ED: End of ejection
EDS: Ehlers-Danlos syndrome
HR: Heart rate
ICAM-1: Intercellular adhesion molecule 1
kEDS: Kyphoscoliotic Ehlers-Danlos syndrome
MCP-1: Monocyte chemoattractant protein 1
Min: Minute
NSAID: Non-steroidal anti-inflammatory drug
PP: Pulse pressure
SD: Standard deviation
VCAM-1: Vascular cell adhesion molecule 1
vEDS: Vascular type of Ehlers-Danlos syndrome
